# The AT-Hook motif as a versatile minor groove anchor for promoting DNA binding of transcription factor fragments†
†Electronic supplementary information (ESI) available: Peptide synthesis, full experimental procedures and analytical data of the peptides and products obtained. See DOI: 10.1039/c5sc01415h



**DOI:** 10.1039/c5sc01415h

**Published:** 2015-05-26

**Authors:** Jéssica Rodríguez, Jesús Mosquera, Jose R. Couceiro, M. Eugenio Vázquez, José L. Mascareñas

**Affiliations:** a Centro Singular de Investigación en Química Biolóxica e Materiais Moleculares (CIQUS) , Departamento de Química Orgánica , Universidade de Santiago de Compostela , 15782 Santiago de Compostela , Spain . Email: joseluis.mascarenas@usc.es ; Fax: +34 981 595 012 ; Tel: +34 981 576541 ext. 14405

## Abstract

We report the development of chimeric DNA binding peptides comprising a DNA binding fragment of natural transcription factors (the basic region of a bZIP protein or a monomeric zinc finger module) and an AT-Hook peptide motif. The resulting peptide conjugates display high DNA affinity and excellent sequence selectivity. Furthermore, the AT-Hook motif also favors the cell internalization of the conjugates.

## Abstract 2

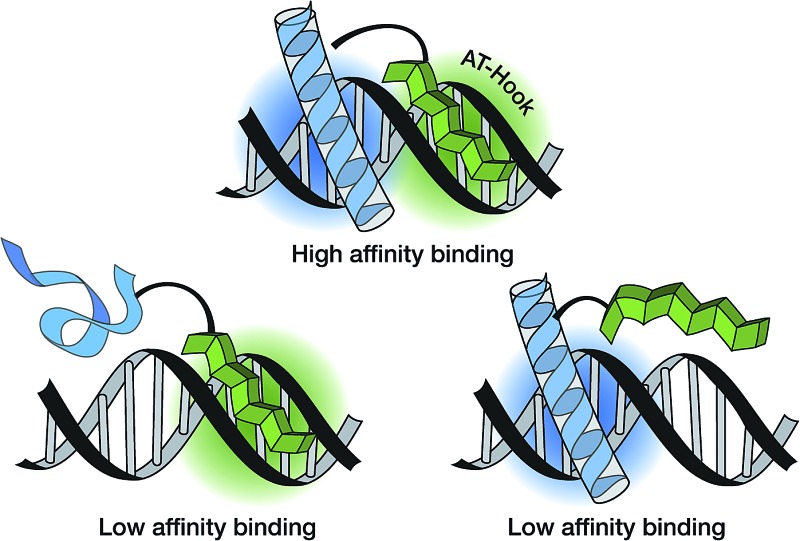
We report the development of chimeric DNA binding peptides comprising a DNA binding fragment of natural transcription factors (the basic region of a bZIP protein or a monomeric zinc finger module) and an AT-Hook peptide motif.

## 

Transcription Factors (TFs) are specialized proteins that, upon recognizing specific DNA sequences, play a key role in the regulation of gene expression.^1^ Therefore, alterations in their activity are at the origin of many diseases, including cancer.^2^ Owing to this relevance, there is a great interest on the development of non-natural DNA binding peptides that can somewhat mimic the DNA binding properties of naturally occurring TFs.^3^ It is well known that although the DNA-binding of TFs is mediated by relatively small peptide motifs, high-affinity DNA recognition requires the full protein domain and in many cases, the concerted action of multiple DNA-binding components.^4^ Therefore, isolated monomeric DNA binding fragments of bZIP or zinc finger TF families do not interact with their target sites with significant affinity. We have previously shown that the DNA binding of these monomeric regions can be restored by conjugation to small molecule minor groove binders, such as distamycin or pentamidine derivatives.^5^ Although these hybrids exhibit interesting recognition properties, they suffer from relatively poor sequence selectivity as a consequence of the intrinsic high affinity of the minor groove binders for A/T-rich DNA sites.^6^ Furthermore, the synthesis of these conjugates requires elaborate multistep procedures, combining solution and solid-phase methods.

In the search for more efficient and selective bivalent DNA binders, we were intrigued by the AT-Hook motif, a naturally occurring cationic short peptide present in HMG-I(Y) eukaryotic nuclear proteins.^7^ It is known that monomeric AT-Hooks bind their target sites with weak affinity (in the millimolar range).^7,8^ However, these proteins attain high DNA-binding affinity thanks to the cooperative action of three appropriately spaced AT-Hook repeats.^9^ NMR and crystallography studies have provided a detailed structural picture of the interaction of the AT-Hook (RKPRGRPKK) with DNA, and have shown that the central Arg-Gly-Arg core of this oligocationic peptide is deeply inserted into the minor groove (Fig. 1, left), while the various lysines in the sequence (R**K**PRGRP**KK**) introduce additional electrostatic contacts with the phosphates of the DNA backbone.^10,11^

**Fig. 1:**
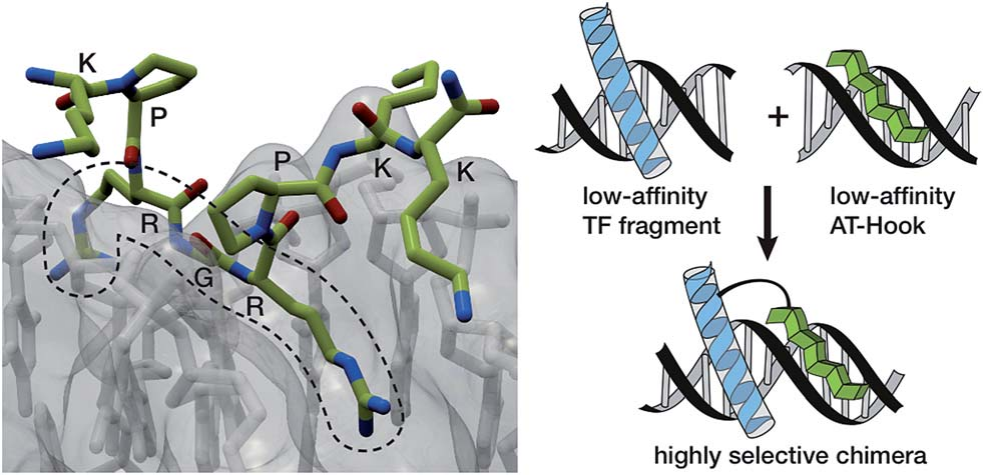
Left: crystal structure of the AT-Hook domain bound to the minor groove of a TTAATTAA sequence, highlighting the insertion of the RGR core sequence into the minor groove. Right: representation of the DNA interaction of a conjugate between a minor groove binder and the DNA recognition helix of a TF with their consensus site. The intrinsic low affinity of the components should provide a good selectivity towards the composite DNA sequence.

The low intrinsic DNA affinity of monomeric AT-Hooks might discourage their application as minor groove anchors to promote the DNA binding of tethered TF fragments. However we envisioned that such low affinity might actually offer an opportunity for achieving greater sequence selectivity, because a high affinity, cooperative interaction should only occur in DNA sequences containing both targeting sites at adjacent positions (Fig. 1, right). Moreover, and importantly, the peptidic nature of the AT-Hook motif should considerably simplify the synthetic access to the conjugates.

Herein we report the design and synthesis of two peptide chimeras consisting of an AT-Hook motif linked to the DNA binding domains of GCN4 and GAGA, selected as representative members of the basic leucine zipper (bZIP) and Cys_2_His_2_ zinc-finger (ZF) families of transcription factors,^1,12^ and demonstrate that these conjugates exhibit excellent DNA binding affinity and selectivity for target specific sites of 8–9 base pairs.

The GCN4/AT-Hook conjugate (**brH**) was based on a GCN4 peptide fragment comprising of residues Asp^226^ to Gln^248^, which has been identified as the shortest peptide that, as a disulfide dimer, retains the specific DNA binding properties of the full GCN4 DNA binding domain.^13^ The design was based on the structures of the GCN4 dimer bound to the AP1 (5′-ATGA(c)TCAT-3′) site,^14^ and the third AT-Hook DNA binding domain of HMG-I(Y), RKPRGRPKK, bound to the PRDII sequence of the IFN-β promoter.^10^ We built a qualitative computer model for the simultaneous interaction of both peptides bound to contiguous sequences of the DNA (see the ESI†). Inspection of this model suggested that the Arg^245^ residue in GCN4, which is oriented towards the adjacent minor groove, was a good candidate for introducing the tether between the two peptides (ESI†). Thus, we synthesized the selected GCN4 basic region fragment containing the mutation Arg^245^ → Lys following standard Fmoc/tBu solid-phase peptide synthesis protocols (**br**, Scheme 1).^15^ The Lys^245^ residue was introduced with its side chain protected by an orthogonal alloc group, which could be selectively removed in the solid phase under Pd catalysis. Subsequent assembly of an O1Pen linker and the AT-Hook sequence, followed by standard deprotection/cleavage steps and reverse-phase HPLC purification, gave the expected peptide **brH** in a good overall yield (approx. 20%, Scheme 1). It should be remarked that the whole synthesis can be completed in one day.

**Scheme 1:**
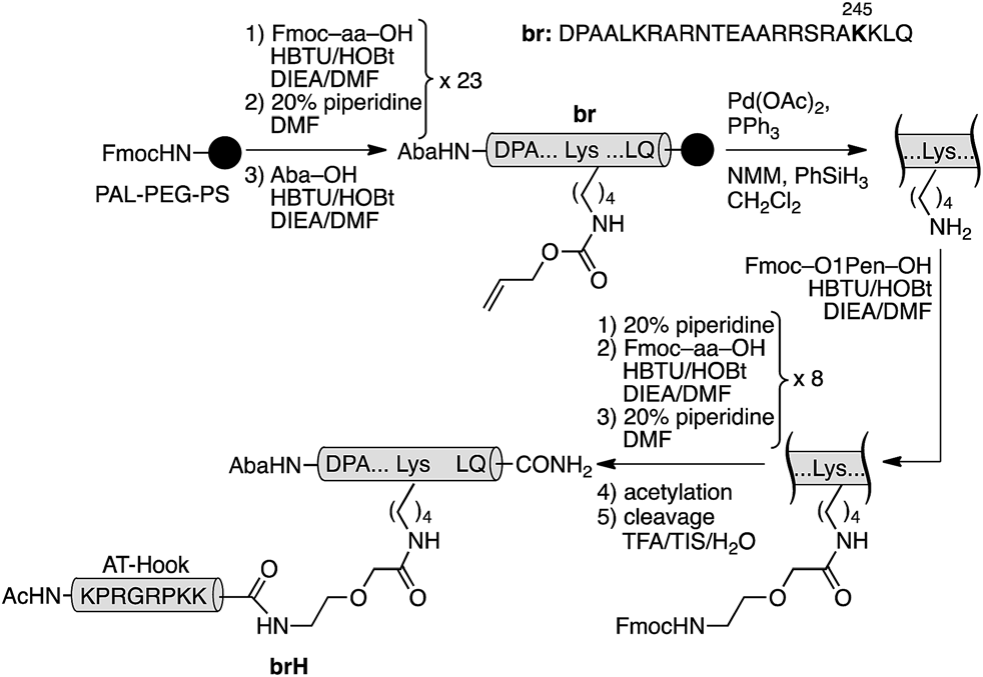
Synthetic route to the GCN4/AT-Hook chimera **brH**. The 4-acetamidobenzoic acid chromophore (Aba) is introduced at the N-terminus of the GCN4 basic region as a spectroscopic reporter.

Having at hand the desired peptidic chimera **brH**, we first studied its DNA binding properties using standard non-denaturing EMSA experiments in polyacrylamide gels.^16^ Thus, the incubation of the ds-oligonucleotide **AP1^hs^·AT**, which contains a composite binding sequence including a AP1 half site (**AP1^hs^**, TCAT) and an A/T-rich tract (**AT**, AATT), with increasing concentrations of **brH** led to a new, slow-migrating band, consistent with the formation of the desired **brH**/**AP1^hs^·AT** complex (Fig. 2a). Importantly, the conjugate **brH** does not elicit retarded bands when incubated with non-target sequences lacking the A/T-rich or the AP1 half site (Fig. 2b and c). This contrasts with previous conjugates with small molecule minor groove binders that also displayed considerable interactions with DNAs containing just A/T-rich sites,^5^ and supports our premise that using low-affinity DNA minor groove binders leads to better selectivities.

**Fig. 2:**
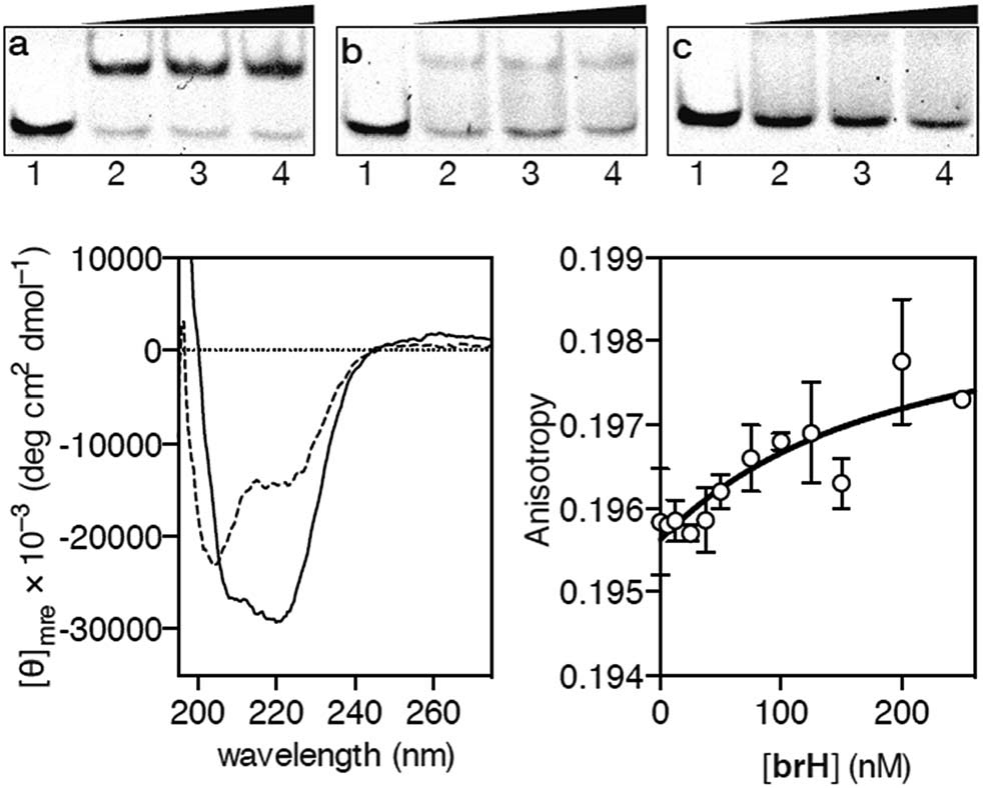
EMSA DNA binding studies results for the conjugate **brH**. (a) Lanes 1–4: [**brH**] = 0, 500, 700, 1000 nM, and 75 nM **AP1^hs^·AT** dsDNA. (b) Lanes 1–4: [**brH**] = 0, 500, 700, 1000 nM, and 75 nM of **AP1^hs^·GC** dsDNA. (c) Lanes 1–4: [**brH**] = 0, 500, 700, 1000 nM, and 75 nM of **GC·AT** dsDNA. Oligonucleotide sequences (only one strand shown): **AP1^hs^·AT**: 5′-CGCGTCAT*AATT*GAGAGCGC-3′; **AP1^hs^·GC**: 5′-CGCGTCATCAGCGAGAGCGC-3′; and **GC·AT**: 5′-GACGG*AATTT*GAGAGCGTCG-3′. Bottom left: circular dichroism of a 5 μM solution (phosphate buffer pH 7.5) of **brH** (dashed line) and the same solution after the addition of 1 equiv. of the target **AP1^hs^·AT** dsDNA (solid lane). The contribution of the DNA to the CD spectrum has been subtracted for clarity. Bottom right: fluorescence anisotropy titration of a 25 nM solution of TMR–**AP1^hs^·AT** in the presence of increasing concentrations of **brH**. The best fit to a 1 : 1 binding model is also shown.

In agreement with the results obtained by the EMSA, circular dichroism (CD) experiments revealed that the addition of 1 equiv. of the target oligonucleotide **AP1^hs^·AT** to a 5 μM solution of **brH** promotes a significant increase in the intensity of the negative bands at 208 and 222 nm, which is consistent with the expected folding of the GCN4 basic region into an α-helix upon insertion in the major groove of the **AP1^hs^** site (Fig. 2, bottom left).^17^ Fluorescence anisotropy titrations using the TMR-labeled dsDNA **AP1^hs^·AT** confirmed that **brH** recognizes the DNA with a high affinity, with an apparent *K*_D_ of ≈28 nM at 20 °C (Fig. 2, bottom right), which is considerably better than the binding constants corresponding to each of the isolated components for their respective targets (in the high micromolar–low millimolar range). Taken together, these results support the formation of a cooperative, bivalent DNA complex at specific composite DNA sites of eight base pairs.

In order to confirm the general applicability of the AT-Hook motif as an effective minor groove DNA anchor for highly efficient and selective interactions, we designed a second chimera based on the DNA-binding domain of the GAGA protein: a monomeric Cys_2_His_2_ zinc finger transcription factor.^18^ As with GCN4, the design of the GAGA hybrid with the AT-Hook motif was based on the structure of the GAGA binding domain complexed with its target h3/h4 (GAGAG) DNA site.^18^ Based on earlier reports that identified the minimal domain required for specific binding,^19^ as well as on our own previous work with GAGA-distamycin hybrids,^20^ we selected a fragment of the DNA binding domain from residues Ser^28^ to Phe^58^ that by itself is incapable of interacting with its target DNA site. The selected peptide sequence was modified to include an appropriate mutation (Arg^44^ → Lys) for tethering the AT-Hook to the αββ core of the transcription factor (see the ESI†). An inspection of the superimposed structures of the AT-Hook and the GAGA DNA binding domain bound to adjacent sites suggested that in this case the conjugate might be better assembled by a chemoselective ligation of fully deprotected peptides. In particular, we designed a synthetic scheme involving the coupling of a reactive bromoacetyl derivative of the AT-Hook with a nucleophilic cysteine attached to the side chain of the Lys^44^ of the zinc finger moiety (Scheme 2). The chemoselective coupling to this thiol could be attained by blocking the other two natural cysteine residues (Cys^36^ and Cys^39^) by coordination to Zn(ii) in the form of the folded zinc finger domain.^21^

**Scheme 2:**
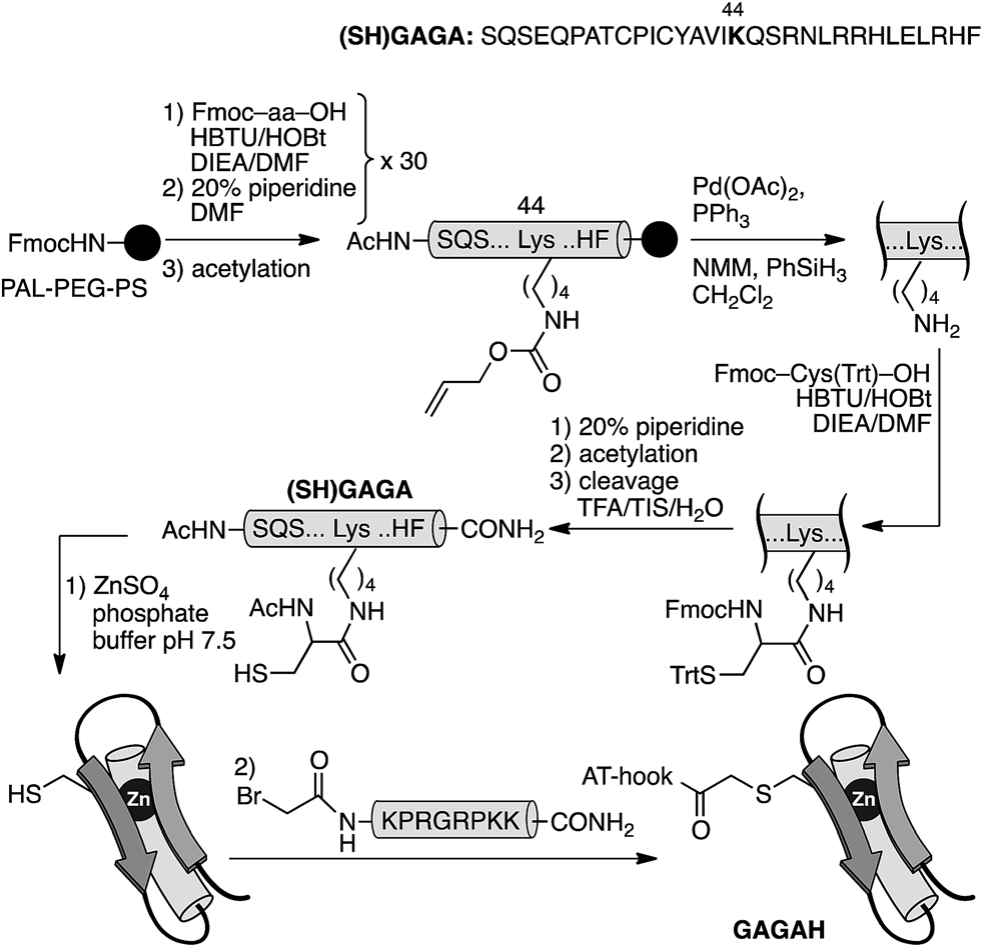
Strategy used for the synthesis of the AT-Hook/GAGA chimera **GAGAH** by the chemoselective modification of the GAGA Cys_2_His_2_ peptide in solution.

The core GAGA peptide containing a Lys^44^ orthogonally protected with an alloc group in its side chain was synthesized following standard Fmoc SPPS procedures. The alloc group was removed under Pd catalysis, and a Cys residue was coupled to the side chain. Fmoc deprotection and acetylation, followed by standard deprotection and cleavage steps led to the expected peptide **(SH)GAGA** (Scheme 2). The AT-Hook sequence was assembled following standard methodology, and capped on its N-terminus with an electrophilic bromoacetyl handle (see the ESI†). Finally, the nucleophilic peptide **(SH)GAGA** and the AT-Hook bromide derivative were coupled in solution in the presence of Zn(ii). The reaction took place at rt in a deoxygenated phosphate buffer (pH 7.5) in the presence of 1.5 equiv. of ZnSO_4_. After 1 h, HPLC-MS analysis showed the formation of the desired product, which was purified by HPLC and identified as the desired conjugate **GAGAH** (see Scheme 2 and the ESI†).

As in the previous case, we assessed the DNA binding of the conjugate using EMSA under non-denaturing conditions. The incubation of a double stranded oligonucleotide containing the target composite binding site for the AT-Hook motif and the GAGA domain (**AT·GAGA**), at rt, with increasing concentrations of the conjugate **GAGAH**, led to the appearance of a new retarded band consistent with the formation of the expected peptide–DNA complex (Fig. 3a). Other oligonucleotides mutated in the GAGA or in the AT-Hook binding site failed to give rise to stable DNA peptide complexes, and indeed barely show the formation of any nonspecific complexes (Fig. 3b and c, respectively). Moreover, fluorescence anisotropy titrations using TMR-labeled dsDNA confirmed the high affinity interaction of the peptide chimera, with an apparent *K*_D_ of 26 nM at 20 °C (Fig. 3, right). The DNA interaction in the presence of an excess of competing calf thymus DNA displays a decreased—but still very significant—*K*_D_ of 42 nM (see the ESI†).

**Fig. 3:**
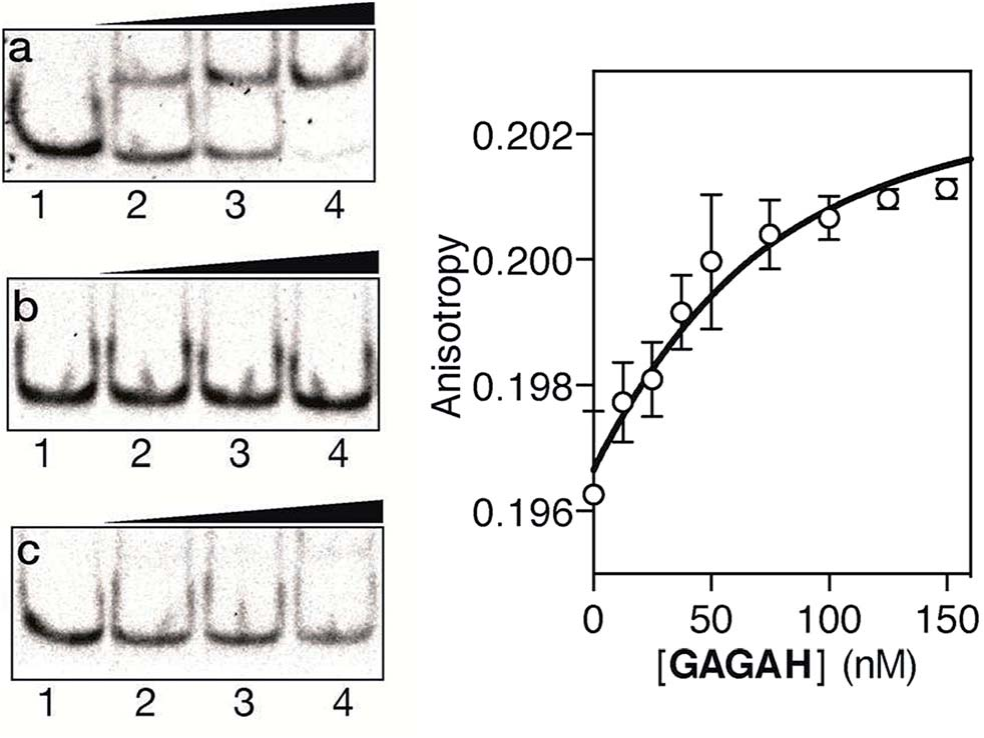
Left: EMSA DNA binding studies results for the conjugate **GAGAH**. (a) Lanes 1–4: [**GAGAH**] = 0, 500, 700, 1000 nM, and 75 nM **AT·GAGA** dsDNA. (b) Lanes 1–4: [**GAGAH**] = 0, 500, 700, 1000 nM, and 75 nM of **AT·CG** dsDNA. (c) Lanes 1–4: [**GAGAH**] = 0, 500, 700, 1000 nM, and 75 nM of **GC·GAGA** dsDNA. Oligonucleotide sequences (only one strand shown): **AT·GAGA**: 5′-CGCGTCAT*AATT*GAGAGCGC-3′; **AT·CG**: 5′-CGCGTCAT*AATT*CGCGACGC-3′; **GC·GAGA**: 5′-CGCGTCAT *CAGC*GAGAGCGC-3′. Right: fluorescence anisotropy titration of a 25 nM solution of TMR–**AT·GAGA** in the presence of increasing concentrations of **GAGAH**. The fit to a 1 : 1 binding model is also shown.

A limitation for the potential biological application of this type of DNA-binding peptide construct might derive from its presumable poor cell internalization. However, it is known that oligocationic peptides have a beneficial effect in cellular transport,^22^ and therefore we decided to check whether the presence of the AT-Hook could enhance the cellular entrance of these conjugates. Thus, as a preliminary test, we incubated mammalian Vero cells with a tetramethylrhodamine (TMR) derivative of **brH**, as well as with a control peptide that lacks the AT-Hook (TMR–**br**, see the ESI†). Remarkably, while TMR–**br** was essentially not internalized,^23^ the incubation of the cells with TMR–**brH** led to a bright emission inside the cells, which was mainly localized in endosomes (Fig. 4).^24^ Therefore it is clear that the presence of the AT-Hook enhances the transport across the cell membrane, which opens the door to the cellular applications of these peptide chimeras.

**Fig. 4:**
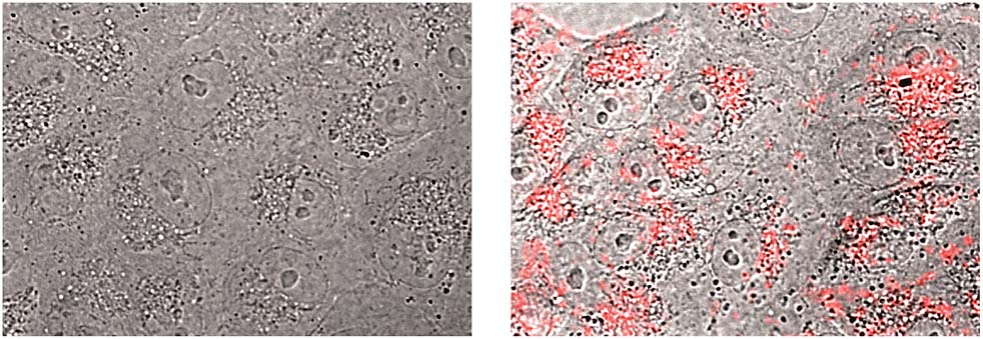
Fluorescence micrographs of Vero cells. The bright field images are superimposed with the red emission channel after incubation with 5 μM TMR–**br** (left) or TMR–**brH** (right) for 30 min at 37 °C.

## Conclusions

Taken together, these results demonstrate that the AT-Hook motif is a synthetically accessible minor groove binder that can be effectively exploited for the straightforward construction of functional DNA binding conjugates containing TF peptide fragments. The resulting bivalent chimeras display excellent DNA recognition properties in terms of affinity and selectivity, properties that rely on the cooperative action of two weakly DNA binding components. This work highlights the role of the hitherto overlooked AT-Hook motif as a DNA binding handle. The synthetic accessibility and nanomolar affinity and selectivity exhibited by these conjugates, together with their good cell transport properties, allows anticipation of potential applications to modulate gene transcription processes.
