# Supplementary material for: The AT-Hook motif as a versatile minor groove anchor for promoting DNA binding of transcription factor fragments
Source: Chem Sci. 2015 May 26;6(8):4767–71. doi: 10.1039/c5sc01415h (PMC4538796; doi:10.1039/c5sc01415h)
Supplement: Supplementary file 1 [file SC-006-C5SC01415H-s001.pdf]

Electronic Supplementary Material (ESI) for Chemical Science.
This journal is © The Royal Society of Chemistry 2015

ESI

The AT-Hook motif as a versatile minor groove anchor for promoting the DNA binding of transcription factor fragments

Jéssica Rodríguez, Jesús Mosquera, Jose R. Couceiro, M. Eugenio Vázquez,* and José L. Mascareñas*

Centro Singular de Investigación en Química Biolóxica e Materiais Moleculares (CIQUS), Departamento de Química Orgánica. Universidade de Santiago de Compostela, 15782 Santiago de Compostela, Spain.

Abbreviations

DEDTC: sodium diethyldithiocarbamate

DIC: *N,N'*-Diisopropylcarbodiimide

EDT: ethanedithiol

HATU: 2-(1H-7-aza-benzotriazol-1-yl)-1,1,3,3-tetramethyluroniumhexafluorophosphate

HBTU: 2-[(1H-benzotriazol-1-yl)-1,1,3,3-tetramethyluronium hexafluorophosphate

TFA: trifluoroacetic acid

TIS: triisopropylsilane

TMR: tetramethylrhodamine dye

General peptide synthesis procedures

All peptide synthesis reagents and amino acid derivatives were purchased from GL Biochem (Shanghai) and Novabiochem; amino acids were purchased as protected Fmoc amino acids with the standard side chain protecting scheme: Fmoc-Ala-OH, Fmoc-Leu-OH, Fmoc-Lys(Boc)-OH, Fmoc-Ser(*t*-Bu)-OH, Fmoc-Glu(O*t*-Bu)-OH, Fmoc-Trp(Boc)-OH, Fmoc-Asn(Trt)-OH, Fmoc-Ile-OH, Fmoc-Thr(*t*-Bu)-OH, Fmoc-Arg(Pbf)-OH, Fmoc-His(Trt)-OH and Fmoc-Asp(O*t*-Bu)-OH except for the orthogonally protected Fmoc-Lys(Alloc)-OH, which was purchased from *Bachem*. All other chemicals were purchased from *Aldrich* or *Fluka*. All solvents were dry and synthesis grade, unless specifically noted.

Peptides were synthesized using an automatic peptide synthesizer from *Protein Tecnologies PS3 PeptideSynthesizer*. Peptide synthesis was performed using standard Fmoc solid-phase method on a PAL–PEG–PS resin (0.19 mmol/g) using HBTU/HOBt (4 equiv) as coupling agent, DIEA as base (6 equiv) and DMF as solvent. The deprotection of the temporal Fmoc protecting group was performed by treating the resin with 20% piperidine in DMF solution for 20 min. For **brH**, after the N-terminal aspartic acid, we coupled p-ABA chromophore.

Side chain deprotection: The resin containing the alloc peptide (75 mg, aprox. 0.015 mmol) was suspended in DMF and shaken for 1 h to ensure a good swelling. Then $Pd(OAc)_2$ (1 mg, 0.3 equiv), 4-methylmorpholine (16 μL, 10 equiv), PPh_3 (6 mg, 1.5 equiv), $PhSiH_3$ (18 μL, 10 equiv) and 1 mL CH_2Cl_2 were added, and the mixture shaken overnight. The resin was then filtered and washed with DMF, DEDTC and DMF again.

Coupling of acetic anhydride: The resin containing the peptide (50 mg, aprox. 0.01 mmol) was suspended in DMF and shaken for 1 h to ensure a good swelling. A solution of acetic anhydride (20 uL, 0.2 mmol, 20 equiv) and DIEA (400 μL, 0.195 M in DMF, 0.08 mmol, 8 equiv) was added. The reaction mixture was shaken for 0.5 h. The resin was washed with DMF (3 ×, 5 min) and DCM (2 ×, 5 min).

Coupling of bromoacetic acid: The resin containing the peptide (50 mg, aprox. 0.01 mmol) was suspended in dichloromethane (DCM) and shaken for 1 h to ensure a good swelling. On the other hand, bromoacetic acid (28 mg, 20 equiv) was dissolved in 630 µL CH_2Cl_2 and cooled to 0 ºC. Diisopropylcarbodiimide (DIC, 16 µL, 10 equiv) was added, and the mixture stirred at 0 ºC for 20 min. The solid was filtered off and the flitrate was added over the resin. The suspension was shaken for 30 min. The resin was then filtered and washed with DCM.

For TMR-**br** and TMR-**brH**, after the N-terminal aminoacid, we coupled 5-(Fmoc-amino)valeric acid as spacer between the peptide and the fluorophore, and finally TMR fluorophore: 5(6)-carboxytetramethylrhodamine was coupled using 3 equiv of the rhodamine (0.15 mmol, 64.5 mg), 3 equiv of HATU and 5 equiv of DIEA 0.2 M in DMF for 60 min.

The cleavage/deprotection step was performed by treatment of the resin-bound peptide for 1.5–2h with the following cleavage cocktails: 940 µL TFA, 25 µL EDT, 25 µL H_2O and 10 µL TIS (1 mL of cocktail / 40 mg resin) for cysteine-containing peptides and 900 µL TFA, 50 µL CH_2Cl_2, 25 µL H_2O and 25 µL TIS (1 mL of cocktail / 40 mg resin) for the rest.

The crude products were purified by RP−HPLC, 4 mL/min, gradient 10 to 50% B over 40 min. (A: H_2O 0.1% TFA, B: CH_3CN 0.1% TFA) and identified as the desired peptides.

High-Performance Liquid Chromatography (HPLC) was performed using an *Agilent 1100* series Liquid Chromatograph Mass Spectrometer system. Analytical HPLC was carried out using a *Eclipse XDB-C18 analytical column (4.6 x 150 mm, 5 µm)*, 1 mL/min, gradient 5 to 75% B over 30 min. Purification of the peptides was performed on a semipreparative *Phenomenex Luna–C18* (250 × 10 mm) reverse-phase column.

Electrospray Ionization Mass Spectrometry (ESI/MS) was performed with an *Agilent 1100* Series LC/MSD VL G1956A model in positive scan mode.

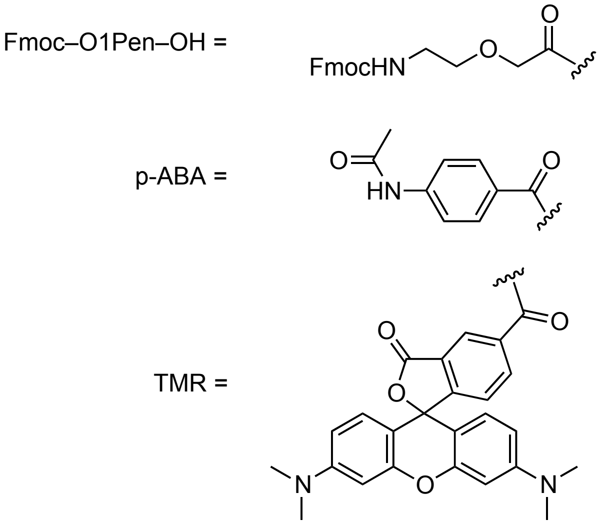

Fig. S1 Structures of Fmoc-O1Pen-OH, p-ABA and TMR.

brH was isolated with an approx. yield of 20%.

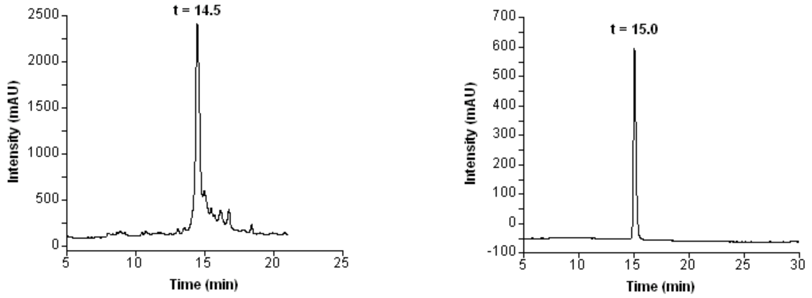

Fig. S2 HPLC chromatogram of: Left) crude residue after the synthesis; Right) purified peptide. Gradient 5 to 75% B over 30 min.

EM–ESI$^+$ (m/z): Calcd. for $C_{165}H_{288}N_{62}O_{45}$: 3858.22. Found: 965.5 $[M+4H]^{4+}$; 772.8 $[M+5H]^{5+}$.

(SH)GAGA was isolated with an approx. yield of 17%.

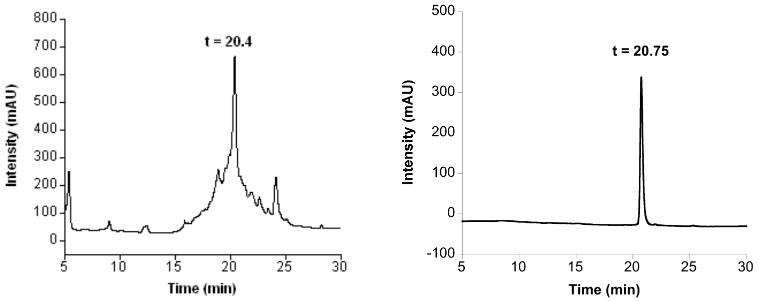

Fig. S3 HPLC chromatogram of: Left) crude residue after the synthesis; Right) purified peptide. Gradient 5 to 75% B over 30 min.

EM–ESI$^+$ (m/z): Calcd. for $C_{166}H_{267}N_{53}O_{48}S_3$: 3869.43. Found: 1289.4 $[M+3H]^{3+}$; 967.7 $[M+4H]^{4+}$.

AT-Hook-Br was isolated with an approx. yield of 19%.

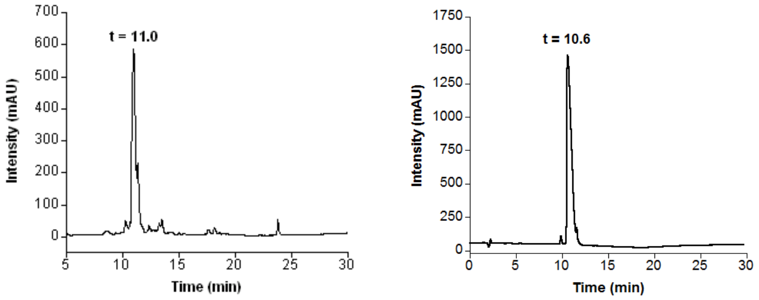

Fig. S4 HPLC chromatogram of: Left) crude residue after the synthesis; Right) purified peptide. Gradient 5 to 75% B over 30 min.

EM–ESI$^+$ (m/z): Calcd. for $C_{44}H_{80}BrN_{17}O_{10}$: 1085.5. Found: 1085.5 $[M+1H]^{1+}$; 544.0 $[M+2H]^{2+}$; 363.1 $[M+3H]^{3+}$.

TMR-**br** was isolated with an approx. yield of 24%.

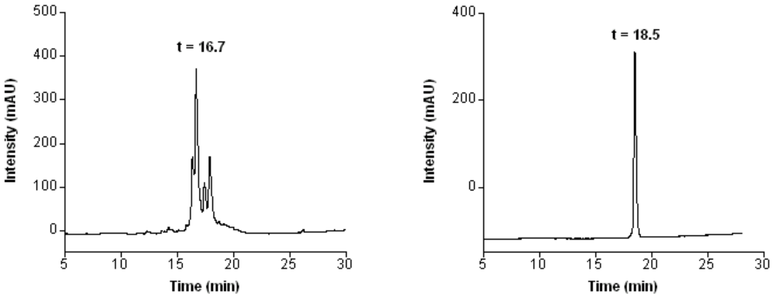

Fig. S5 HPLC chromatogram of: Left) crude residue after the synthesis (gradient 10 to 50% B over 40 min); Right) purified peptide (gradient 5 to 75% B over 30 min).

EM–ESI$^+$ (m/z): Calcd. for $C_{142}H_{230}N_{47}O_{38}$: 3201.64. Found: 1068.1 $[M+3H]^{3+}$; 801.5 $[M+4H]^{4+}$; 641.3 $[M+5H]^{5+}$; 534.2 $[M+6H]^{6+}$.

TMR-**brH** was isolated with an approx. yield of 15%

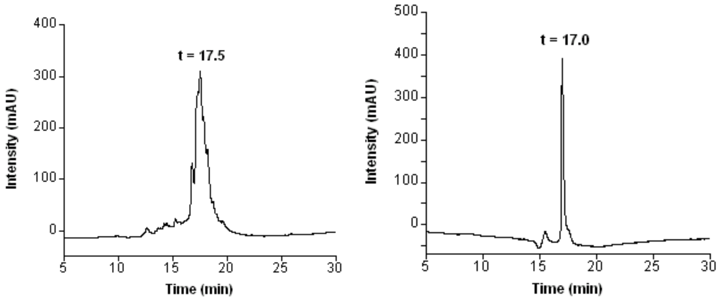

Fig. S6. HPLC chromatogram of: Left) crude residue after the synthesis; Right) purified peptide. Gradient 5 to 75% B over 30 min.

EM–ESI$^+$ (m/z): Calcd. for $C_{184}H_{309}N_{64}O_{47}$: 4169.90. Found: 1042.4 $[M+4H]^{4+}$; 834.7 $[M+5H]^{5+}$; 695.3 $[M+6H]^{6+}$.

Coupling of (SH)GAGA and AT-hook-Br:

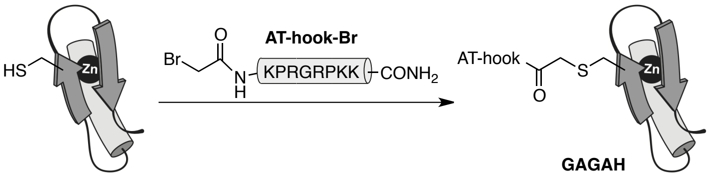

(SH)GAGA (0.1 mg, $3x10^{-5}$ mmol) was dissolved in desoxygenated phosphate buffer (130 µL, 10 mM, pH = 7.5). A $ZnSO_4$ solution ($4.5x10^{-5}$ mmol, 9 µL of a 5 mM solution in Milli-Q water, 1.5 equiv) was added, and the mixture was stirred at room temperature for 10 minutes under argon atmosphere. **AT-Hook-Br** ($6x10^{-5}$ mmol, 5 µL of a 12 mM solution in desoxygenated phosphate buffer, 2 equiv) was added, and the solution was stirred again at room temperature for 1 hour under argon atmosphere.

The product was purified by RP−HPLC, 4 mL/min, gradient 10 to 50% B over 40 min. (A: H_2O 0.1% TFA, B: CH_3CN 0.1% TFA, retention time = 20.4 min), and identified as **GAGAH** by mass spectrometry.

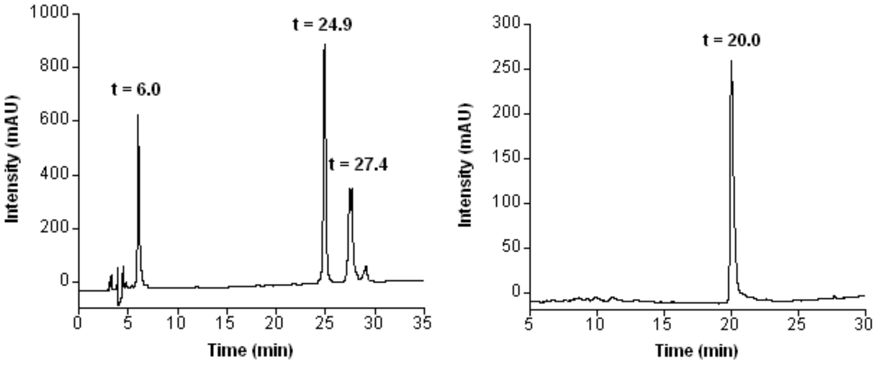

Left) HPLC chromatogram of the crude residue after the synthesis (gradient 5 to 75% B over 30 min).

t = 6.0 starting **AT-Hook-Br**.

t = 24.9 **GAGAH**. **EM–ESI$^+$ (m/z):** Calcd. for $C_{210}H_{346}N_{70}O_{58}S_3$: 4872.54. Found: 1218.9 $[M+4H]^{4+}$; 975.3 $[M+5H]^{5+}$; 813.0 $[M+6H]^{6+}$.

t = 27.4 starting **(SH)GAGA**.

Right: HPLC chromatogram of the purified peptide (gradient 10 to 50% B over 40 min**).**

t = 20.0 **GAGAH**. **EM–ESI$^+$ (m/z):** Calcd. for $C_{210}H_{346}N_{70}O_{58}S_3$: 4872.54. Found: 1218.9 $[M+4H]^{4+}$; 975.3 $[M+5H]^{5+}$; 813.0 $[M+6H]^{6+}$.

Superpositions

This was carried out with MacPymol using the references PDB ID: 1YSA for GCN4, 3UXW for AT-Hook and 1YUI for GAGA.

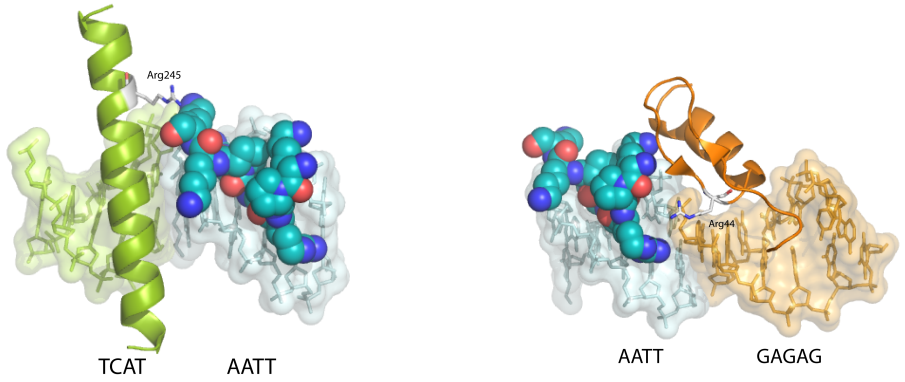

Fig. S7 Model of the simultaneous interaction of: Left) **br** (green) and **AT-Hook**; Right) **(SH)GAGA** (orange) and **AT-Hook**.

Oligonucleotide sequence

Double stranded (only one strand is shown) oligonucleotides used for EMSA experiments with conjugates **brH** and **GAGAH** were supplied by *Thermo Fischer* and their sequences were:

**AP1^{hs}•AT and AT•GAGA**	5´-CGCG**TCAT***AATT*GAGAGCGC-3´
**AT•CG**	5´-CGCG**TCAT***AATT*CGCGACGC-3´
**AP1^{hs}•GC and GC•GAGA**	5´-CGCG**TCAT**CAGCGAGAGCGC-3´
**GC•AT**	5´-GACGG*AATTT*GAGAGCGTCG-3´

EMSA experiments

EMSAs were performed with a BioRad Mini Protean gel system, powered by an electrophoresis power supplies PowerPac Basic model, maximum power 150 V, frequency 50-60 Hz at 140 V (constant V). Binding reactions were performed over 30 min in 18 mM Tris (pH 7.5), 90 mM KCl, 1.8 mM $MgCl_2$, 1.8 mM EDTA, 9% glicerol, 0.11 mg/mL BSA and 2.2% de NP–40 for **brH**, and 18 mM Tris-HCl (pH 7.5), 90 mM KCl, 1.8 mM $MgCl_2$, 0.2 mM TCEP, 9% glycerol, 0.11 mg/mL BSA, 2.2% NP-40 and 0.02 mM of $ZnCl_2$ for **GAGAH**. In the experiments we used 75 nM of the ds−DNAs and a total incubation volume of 20 uL. After incubation for 30 min products were resolved by PAGE using a 10% non-denaturing polyacrylamide gel and 0.5x TBE buffer for 40 min at 20 ºC, and analyzed by staining with SyBrGold (Molecular Probes: 5 μL in 50 mL of 1x TBE) for 10 min. and visualized by fluorescence.

5x TBE buffer: 0.445M Tris, 0.445 M Boric acid, 0.010 M EDTA (pH 8.0) for **brH** and 0.445M Tris, 0.445 M Boric acid for **GAGAH**.

Fluorescence Anisotropy

Measurements were made with a Jobin-Yvon Fluoromax-3, (DataMax 2.20) coupled to a Wavelength Electronics LFI−3751 temperature controller, using the following settings: integration time: 2.0 s; excitation slit width: 5.0 nm; emission slit width: 20.0 nm; excitation wavelength 559 nm; emission wavelength 585 nm.

TMR-AT•GAGA (TMR-5´-CGCG**TCAT***AATT*GAGAGCGC-3´, one strand shown) (5μL, 5 μM) was added to 995 μL of Tris-HCl buffer 20 mM pH 7.5, 100 mM NaCl, 0.02 mM of $ZnCl_2$, and the anisotropy was measured. Aliquots of a stock solution in water of **brH** or **GAGAH** (12.5 μM) were successively added to this solution, and the anisotropic value was recorded after each addition.

In the case of GAGAH, we also made the experiment in the presence of calf thymus DNA (50 μM in base pairs).

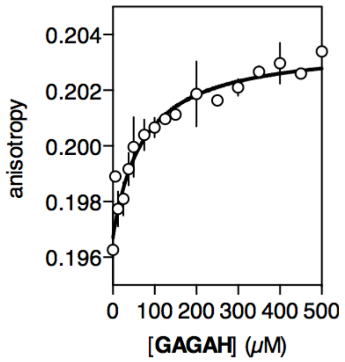

Fig. S8 Plot of the anisotropic values of TMR-**AT•GAGA** in the presence of calf thymus DNA recorded at 585 nm against the total concentration of **GAGAH**, and best-fit curve to a 1:1 binding mode with an estimated K_D of 42 ± 8 nM. Average of three replicates.

CD measurements were made with a Jasco-715 coupled with a thermostat Nestlab RTE-111. The settings used were: Acquisition range: 300-195nm; band width: 2.0 nm; resolution: 0.2 nm; accumulation: 5 scans; sensitivity 10 mdeg; response time: 0.25 s, speed: 100 nm/min. Measurements were made in a 2 mm cell at 20 ºC. Samples contained 10 mM phosphate buffer pH 7.5 and 100 mM of NaCl, 5 μM peptide and 5 μM of corresponding dsDNA (when present). The mixtures were incubated for 5 min before registering. The CD spectra of the peptide (when measured in the presence of DNA) was calculated as the difference between the spectrum of the peptide/DNA mixture and the measured spectrum of a sample of the DNA oligonucleotide. The spectra are the average of 5 scans and were processed using the “smooth” macro implemented in the program *Kaleidagraph* (v 3.5 by Synergy Software).

Cellular internalization studies

Cells growing on glass coverslips were incubated in PBS containing 5 μM of TMR-**br** or TMR-**brH** for 30 min. Then they were washed twice with PBS and observed *in vivo* in a fluorescence microscope equipped with adequate filters and differential interference contrast (DIC) microscopy. Digital pictures of the different samples were taken under identical conditions of gain and exposure.

The assay was carried with three different cell lines: monkey Vero cells, A549 human lung carcinoma cells and HeLa cells.

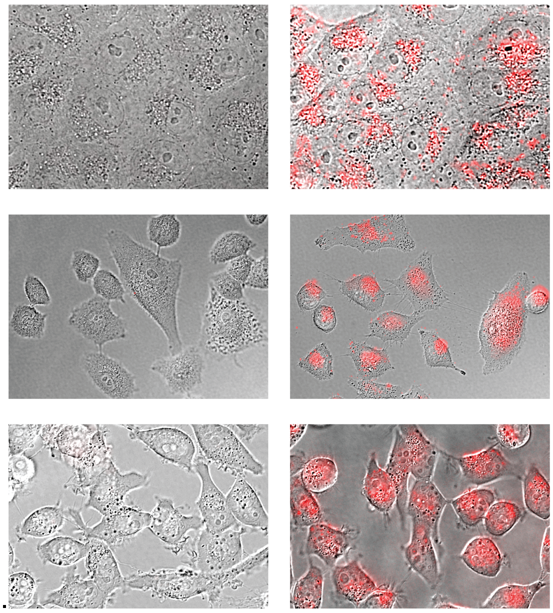

Fig. S9 Fluorescence micrographies. Brightfield images are superimposed to the red emission channel after incubation of cells with 5 μM TMR-**br** (left) or TMR–**brH** (right) for 30 min at 37 ºC. Top row: Vero cells (also included in the main manuscript); Middle row: same experiment with A549 human lung carcinoma cells. Bottom row: same experiment with HeLa cells.
